# A Basic Intervention to Improve Compliance With Thromboprophylaxis in Patients With Neck of Femur Fracture

**DOI:** 10.31486/toj.20.0093

**Published:** 2021

**Authors:** Vinay Kumar Gupta, Keshav Kumar Gupta, Ranjodh Singh Sanghera, Sreenadh Gella

**Affiliations:** ^1^ Department of Gastroenterology, Sandwell and West Birmingham NHS Trust, Sandwell General Hospital, Birmingham, UK; ^2^ Ear, Nose, and Throat Department, Sandwell and West Birmingham NHS Trust, Sandwell General Hospital, Birmingham, UK; ^3^ Emergency Department, Sandwell and West Birmingham NHS Trust, Sandwell General Hospital, Birmingham, UK; ^4^ Department of Orthopedics, Sandwell and West Birmingham NHS Trust, Sandwell General Hospital, Birmingham, UK

**Keywords:** *Fracture–bone*, *hip*, *orthopedics*, *prophylaxis*, *thromboembolism*, *venous thrombosis*

## Abstract

**Background:** Venous thromboembolism (VTE) is a significant complication following orthopedic intervention for neck of femur fracture. Our aim was to evaluate compliance with The National Institute for Health and Care Excellence guidance surrounding VTE prophylaxis before and after a brief intervention in an orthopedic department at a district general hospital.

**Methods:** A 2-cycle quality improvement project was conducted. The primary outcome measure was whether adequate thromboprophylaxis was appropriately prescribed. For the intervention between the 2 cycles, posters were placed in key prescribing areas of all orthopedic wards.

**Results:** In cycle 1, 63 (76.8%) patients were correctly prescribed enoxaparin, and 14 (17.1%) were prescribed other anticoagulants, leaving 5 patients (6.1%) who did not receive thromboprophylaxis for no apparent reason. In cycle 2, 56 (87.5%) patients were correctly prescribed enoxaparin, and the remaining patients were covered with alternative therapies.

**Conclusion:** Small but basic interventions can lead to improvements in VTE prophylaxis prescribing. Future focus should be on implementing similar interventions across hospitals.

## INTRODUCTION

Fragility and hip fractures are increasingly common because of the aging population^1^ and represent a significant health care burden because of the high morbidity and mortality associated with each fracture. The National Institute for Health and Care Excellence (NICE) estimates that approximately 65,000 patients sustain a hip fracture each year, with an annual cost to the United Kingdom of £1 billion.^2,3^ NICE guidance also suggests that 10% of patients who experience neck of femur (NOF) fractures die within 1 month, and 33% die within 12 months.^3^

Venous thromboembolism (VTE) is one of the most dangerous and significant complications of hip fractures and encompasses deep vein thrombosis (DVT) and pulmonary embolism. Estimates suggest that more than half a million deaths in Europe can be attributable to VTE every year, and 1.66 million cases of nonfatal symptomatic VTE are diagnosed in Europe per year.^4^

Orthopedic patients who have an NOF fracture fixation are at high risk for VTE development, generally because of a multitude of patient factors (age >60 years, obesity, multiple comorbidities) and hospital factors (surgery to the lower limb, postoperative immobility).^5,6^ In addition, VTE risk factors have been shown to be cumulative.^7^

Randomized clinical trials have shown that the rate of confirmed DVT in patients who do not receive VTE prophylaxis following orthopedic surgery are approximately 50%.^8^ Furthermore, these patients are still at risk following discharge. VTE is the most common cause for readmission after a total hip replacement and can occur up to 3 months postprocedure.^9^ The use of VTE prophylaxis in this patient group has been shown to reduce the incidence of DVTs and pulmonary embolisms.^2,10^

As a result, NICE has published guidance stating VTE prophylaxis should be offered for 28 to 35 days postoperatively to all patients with fragility hip fractures, provided the risk of VTE outweighs the risk of bleeding.^2^ NICE recommends the use of low molecular weight heparin (LMWH) or fondaparinux starting 6 to 12 hours postoperatively. Despite this recommendation and similar guidance in other countries, the use of VTE prophylaxis is low (52.4%-70%).^11,12^

In the British system of hospital medicine, junior doctors generally rotate every 4 to 6 months and therefore may not be initially familiar with all the guidelines in each department they work in. The aim of this study was to investigate compliance with the NICE VTE prophylaxis guidance in our hospital, to conduct a quality improvement intervention, and to determine compliance after the intervention. The standard was that 100% of patients should receive adequate thromboprophylaxis.

## METHODS

A 2-cycle quality improvement project was conducted to assess compliance with the NICE thromboprophylaxis guidance. During the first cycle, all patients presenting with an NOF fracture during a 3-month period (April through June 2018) at Sandwell General Hospital were identified by the coding department. Case notes and relevant prescriptions were retrospectively analyzed. Data were extracted for patient age, sex, date of admission, primary procedure performed, and if the patient was on any long-term anticoagulation for any other indication (eg, apixaban for atrial fibrillation).

Excluded were patients with pelvic fractures; patients with thromboprophylaxis contraindications (eg, high bleeding risk); and patients who were not operated on, who died during their inpatient stay, or who self-discharged or were still inpatients at the time of data collection. Additionally, patients with incorrect coding or wrong patient identifiable data were excluded.

The primary outcome measure was whether adequate thromboprophylaxis was appropriately prescribed. The secondary outcome measure was whether thromboprophylaxis was specified in the operation note.

Following the first cycle of data collection and analysis, an intervention was conducted to evaluate whether a change would be seen in rates of thromboprophylaxis prescribing. Fifteen posters were placed in key prescribing areas (doctors’ office, ward bays, and next to all computers) of both orthopedic wards in the trust that stated, “ALL NOF FRACTURE PATIENTS NEED 28-35 DAYS OF THROMBOPROPHYLAXIS UNLESS CONTRAINDICATED.” The posters remained in place for a minimum of 3 months before the second data collection cycle with the same methodology was repeated for another 3-month period (August through October 2018) at the same centers.

Collected data were categorical and analyzed using Excel, version 16.31 (Microsoft Corp). Basic descriptive statistics were used to analyze the data.

## RESULTS

During the first data collection cycle, 95 patients were admitted with NOF fractures. Eighty-two patients were included in the analysis after 13 patients were excluded: 5 were still inpatients at the time of data collection, 3 died as inpatients, 3 were not operated on, 1 had a high bleeding risk, and 1 self-discharged.

During the second data collection cycle, 78 patients were admitted with NOF fractures. Sixty-four patients were included in the analysis after exclusion of 14 patients: 7 were still inpatients at time of data collection, 2 died as inpatients, 3 were not operated on, and 2 had incorrect patient identifiable information in the database. The most commonly performed method of fixation was hemiarthroplasty in cycles 1 (n=31, 37.8%) and 2 (n=22, 34.4%). Table 1 summarizes the baseline characteristics, procedures, and prior long-term anticoagulation status for patients in each cycle.

**Table 1.** Baseline Characteristics, Long-Term Anticoagulation Status, and Procedures for Patients in the Data Collection Cycles

Variable	Data Collection Cycle 1 n=82	Data Collection Cycle 2 n=64
Male	24 (29.3)	20 (31.3)
Age ≥65 years	76 (92.7)	61 (95.3)
Prior long-term anticoagulation	12 (14.6)	6 (9.4)
Procedure		
Hemiarthroplasty	31 (37.8)	22 (34.4)
Intramedullary nailing	12 (14.6)	18 (28.1)
Dynamic hip screw	29 (35.4)	17 (26.6)
Total hip replacement	10 (12.2)	7 (10.9)

Note: Data are presented as n (%).

Analysis of discharge prescriptions in cycle 1 revealed that 63 (76.8%) patients were correctly prescribed enoxaparin, an LMWH, according to hospital guidance (enoxaparin is used as the primary LMWH). Analysis of the 19 patients who were not prescribed enoxaparin revealed that 14 (17.1%) were covered by other anticoagulants: 12 patients were on long-term anticoagulation, 1 patient was started on apixaban as an inpatient, and 1 patient was prescribed a direct-acting oral anticoagulant (DOAC) postoperatively for thromboprophylaxis as specified by the operative note. Five patients (6.1%) were not prescribed thromboprophylaxis for no apparent reason.

In cycle 2, 56 (87.5%) patients were correctly prescribed enoxaparin. Of the remaining 8 patients, all were adequately covered by anticoagulation: 6 patients were on long-term anticoagulation, and 2 patients were started on anticoagulants as inpatients. Therefore, all 64 patients (100%) received adequate thromboprophylaxis in the second cycle. Anticoagulant coverage and prescribing are summarized in Table 2.

**Table 2.** Postoperative Anticoagulation Status for Patients With Neck of Femur Fractures in the Data Collection Cycles

Anticoagulation Status	Data Collection Cycle 1 n=82	Data Collection Cycle 2 n=64
Low molecular weight heparin (enoxaparin) prescribed	63 (76.8)	56 (87.5)
Covered with anticoagulation at discharge	14 (17.1)	8 (12.5)
Given DOAC for 28 days	1	0
Prior long-term anticoagulation	12	6
DOAC	11	5
Warfarin	1	1
Started on anticoagulation as an inpatient	1	2
Not covered with anticoagulation at discharge	5 (6.1)	0 (0.0)

Note: Data are presented as n (%) or as n only.

DOAC, direct-acting oral anticoagulant.

Examination of the operation notes showed that 68 notes (82.9%) specified the necessity of thromboprophylaxis in the postoperative plan in cycle 1, and 53 notes (82.8%) included this information in cycle 2.

## DISCUSSION

This study shows that a basic intervention can improve compliance to reach national standards. Through a small change, we were able to improve compliance from 93.9% to 100%. When considering the large number of procedures performed each year, this relative increase of 6.1% has the potential to affect a large number of patients.^8^

The economic burden of VTE is well recognized. Patients who incur this postsurgical complication often require readmission, and both length of hospital stay and cost of inpatient care are significantly increased as a result.^13,14^ The economic burden is further reflected by the National Health Service Commissioning Board policies that reward trusts if they conduct VTE assessments on >95% of adult inpatients.^15^

The fact that the specification of thromboprophylaxis in the operation notes remained static between cycles (82.9% vs 82.8%) suggests that the lack of a specific intervention for operative notes produced no change. Consequently, this outcome acts as a relative control for the study; an outcome measure with no intervention showed no change compared to the primary outcome that did show a change with an intervention.

Our findings add to the results of similar studies conducted in 2013 and 2014.^16,17^ For their interventions, Sinha et al placed a small prescribing reminder on the back of junior doctor work phones,^16^ and Watts and Grant asked consultants to remind junior doctors of prescribing guidance in addition to poster reminders.^17^ Both studies demonstrated an improvement in thromboprophylaxis prescribing following their interventions. However, their sample sizes were smaller (n<50) than this study, thus limiting the validity of conclusions drawn. Furthermore, no analysis in either study regarded patients on other forms of anticoagulation such as DOACs.

Although the NICE guidance emphasises thromboprophylaxis with LMWH or fondaparinux, evidence is emerging regarding DOACs. A meta-analysis in 2012 compared DOACs to enoxaparin for thromboprophylaxis following total hip or knee replacements.^18^ Sixteen trials representing 38,747 patients were included in the meta-analysis. The study concluded that efficacy and safety did not differ significantly overall; however, bleeding tendencies were higher with the DOACs compared to enoxaparin. This dataset represents a robust trial design with minimal evidence of publication bias. However, an important note is that patients included in clinical trials are often not fully representative of the general population, generally because of the exclusion criteria, such as severe renal disease or concomitant use of nonsteroidal anti-inflammatory drugs. The comparisons may therefore not represent a true VTE or bleeding risk.^19^

Although our study was conducted during 2 different time periods, the results are limited by the low sample size. However, compared to similar studies,^16,17^ our numbers are larger. Our findings therefore add to the existing data showing that simple interventions can help to improve doctor awareness regarding national guidance. Furthermore, our samples represent all patients admitted to the orthopedic unit with an NOF fracture during two 3-month periods at a district general hospital and therefore are likely representative of acute orthopedic admissions.

Although we demonstrated an increase in thromboprophylaxis prescribing rates, we cannot be sure if a difference in actual VTE rates occurred between the patient groups. Patients with VTE are admitted under the medical, not surgical, team in the United Kingdom. Therefore, accurately gathering data on admissions because of postoperative VTE is difficult. Furthermore, patients who develop a large VTE (eg, saddle pulmonary embolus) may not survive in the community and therefore will not be included in any analysis of postoperative VTE hospital admissions. Finally, whether any VTE sustained in a postoperative period is directly related to a failure of thromboprophylaxis prescribing is difficult to prove. Further research could build on our study by comparing rates of postoperative VTE as the primary outcome measure.

Our study was conducted at a single center that is not a major trauma unit. To gain a better understanding of thromboprophylaxis prescribing, and therefore determine what procedures can be changed with interventions, more centers in different regions would have to be examined. These measures would add to the power of the study and improve the validity of the conclusions. Finally, patients could have been missed if they were managed on nonorthopedic wards. These patients would not have been included in the dataset, and the junior doctors involved in their prescribing would not have received the intervention. Perhaps an extension of this intervention would be to include it in hospital inductions for new doctors so that all junior doctors are aware. Regardless, our intervention is easily reproducible and applicable to hospitals across the world.

The intervention remains in place at our hospital, and because of the improvements in compliance, we plan to conduct another data collection cycle to determine if the improved prescribing outcome is maintained.

Given the global shift toward digital patient care systems and online prescribing, we feel the next step to maintain high compliance rates of VTE prescribing should be centered on creating specific automated prompts or prescribing templates that help the discharging doctor remember to consider VTE prophylaxis on discharge.

## CONCLUSION

Patients who have sustained an NOF fracture fall into the high-risk category for developing serious surgical complications as a result of VTE. Despite the well-documented risk of VTE in postoperative orthopedic patients, failure to adhere to the NICE thromboprophylaxis guidance can leave these patients unprotected from this avoidable risk. The importance of small but basic interventions cannot be underestimated, as such an intervention showed a benefit in prescribing rates in our dataset. We believe a future focus should be on implementing long-lasting interventions in all surgical wards to improve VTE prophylaxis prescribing rates. This study shows that this goal is achievable with the use of small prompts to help achieve the standards established by NICE.

## Notes

*This article meets the Accreditation Council for Graduate Medical Education and the American Board of Medical Specialties Maintenance of Certification competencies for Patient Care, Medical Knowledge, and Practice-Based Learning and Improvement.*

## References

[1] Yassa R, Khalfaoui MY, Hujazi I, Sevenoaks H, Dunkow P. Management of anticoagulation in hip fractures: a pragmatic approach. EFORT Open Rev. 2017;2(9):394-402. doi: 10.1302/2058-5241.2.160083 29071124 PMC5644423

[2] Venous thromboembolism in over 16s: reducing the risk of hopsital-aquired deep vein thrombosis or pulmonary embolism. National Institute for Health and Care Excellence. March 21, 2018. Updated August 13, 2019. Accessed February 2, 2021. https://www.nice.org.uk/guidance/ng89/chapter/Recommendations#interventions-for-people-having-orthopaedic-surgery

[3] National Clinical Guidance Centre. The management of hip fractures in adults. National Institute for Health and Care Excellence. 2011. Accessed February 4, 2021. https://www.nice.org.uk/guidance/cg124/evidence/full-guideline-pdf-183081997

[4] Cohen AT, Agnelli G, Anderson FA, et al; VTE Impact Assessment Group in Europe (VITAE). Venous thromboembolism (VTE) in Europe. The number of VTE events and associated morbidity and mortality. Thromb Haemost. 2007;98(4):756-764. 17938798 10.1160/TH07-03-0212

[5] Prevention and management of venous thromboembolism. Scottish Intercollgeiate Guidance Network. October 2014. Accessed February 2, 2021. https://www.sign.ac.uk/our-guidelines/prevention-and-management-of-venous-thromboembolism/

[6] Anderson FA Jr, Spencer FA. Risk factors for venous thromboembolism. Circulation. 2003;107(23 Suppl 1):I9-I16. 12814980 10.1161/01.CIR.0000078469.07362.E6

[7] Dorfman M, Chan SB, Maslowski C. Hospital-acquired venous thromboembolism and prophylaxis in an integrated hospital delivery system. J Clin Pharm Ther. 2006;31(5):455-459. doi: 10.1111/j.1365-2710.2006.00764.x 16958823

[8] Geerts WH, Pineo GF, Heit JA, Prevention of venous thromboembolism: the Seventh ACCP Conference on Antithrombotic and Thrombolytic Therapy. Chest. 2004;126(3 Suppl):338S-400S. doi: 10.1378/chest.126.3_suppl.338S 15383478

[9] White RH, Romano PS, Zhou H, Rodrigo J, Bargar W. Incidence and time course of thromboembolic outcomes following total hip or knee arthroplasty. Arch Intern Med. 1998;158(14):1525-1531. doi: 10.1001/archinte.158.14.1525 9679793

[10] McNamara I, Sharma A, Prevost T, Parker M. Symptomatic venous thromboembolism following a hip fracture. Acta Orthop. 2009;80(6):687-692. doi: 10.3109/17453670903448273 19968601 PMC2823318

[11] Cohen AT, Tapson VF, Bergmann JF, et al; ENDORSE Investigators. Venous thromboembolism risk and prophylaxis in the acute hospital care setting (ENDORSE study): a multinational cross-sectional study. Lancet. 2008;371(9610):387-394. doi: 10.1016/S0140-6736(08)60202-0 18242412

[12] Flevas DA, Megaloikonomos PD, Dimopoulos L, Mitsiokapa E, Koulouvaris P, Mavrogenis AF. Thromboembolism prophylaxis in orthopaedics: an update. EFORT Open Rev. 2018;3(4):136-148. doi: 10.1302/2058-5241.3.170018 29780621 PMC5941651

[13] Baser O, Supina D, Sengupta N, Wang L, Kwong L. Clinical and cost outcomes of venous thromboembolism in Medicare patients undergoing total hip replacement or total knee replacement surgery. Curr Med Res Opin. 2011;27(2):423-429. doi: 10.1185/03007995.2010.545940 21192759

[14] Ollendorf DA, Vera-Llonch M, Oster G. Cost of venous thromboembolism following major orthopedic surgery in hospitalized patients. Am J Health Syst Pharm. 2002;59(18):1750-1754. doi: 10.1093/ajhp/59.18.1750 12298113

[15] NHS Commissioning Board. Commissioning for quality and innovation (CQUIN): 2013/14 guidance. Draft – December 2012. National Health Service. December 2012. Accessed February 4, 2021. https://www.england.nhs.uk/wp-content/uploads/2012/12/cquin-guidance.pdf

[16] Sinha P, Najefi AA, Hambidge J. A simple measure to improve the rates of thromboprophylaxis prescription post surgical fixation of neck of femur fractures in a district general hospital. BMJ Qual Improv Rep. 2014;3(1):u202991.w1956. doi: 10.1136/bmjquality.u202991.w1956 PMC4645912 26734300

[17] Watts L, Grant D. Venous thromboembolism (VTE) risk assessment and prophylaxis in acute orthopaedic admissions: improving compliance with national guidelines. BMJ Qual Improv Rep. 2013;2(2):u202229.w1118. doi: 10.1136/bmjquality.u202229.w1118 PMC4663810 26734209

[18] Gómez-Outes A, Terleira-Fernández AI, Suárez-Gea ML, Vargas-Castrillón E. Dabigatran, rivaroxaban, or apixaban versus enoxaparin for thromboprophylaxis after total hip or knee replacement: systematic review, meta-analysis, and indirect treatment comparisons. BMJ. 2012;344:e3675. doi: 10.1136/bmj.e3675 22700784 PMC3375207

[19] Deitelzweig SB, Lin J, Lin G. Preventing venous thromboembolism following orthopedic surgery in the United States: impact of special populations on clinical outcomes. Clin Appl Thromb Hemost. 2011;17(6):640-650. doi: 10.1177/1076029611404215 21593017

